# Supplementary material for: A fault-intrusion-tolerant system and deadline-aware algorithm for scheduling scientific workflow in the cloud
Source: PeerJ Comput Sci. 2021 Nov 2;7:e747. doi: 10.7717/peerj-cs.747 (PMC8576554; doi:10.7717/peerj-cs.747)
Supplement: Supplemental Information 6 [file peerj-cs-07-747-s006.zip › Myproject1.1/docs/org/cloudbus/cloudsim/provisioners/PeProvisionerSimple.html]

PeProvisionerSimple


JavaScript is disabled on your browser.


- Overview
- Package
- Class
- Use
- Tree
- Deprecated
- Index
- Help

- Prev Class
- Next Class

- Frames
- No Frames

- All Classes

- Summary:
- Nested |
- Field |
- Constr |
- Method

- Detail:
- Field |
- Constr |
- Method


org.cloudbus.cloudsim.provisioners

## Class PeProvisionerSimple

- java.lang.Object
- - org.cloudbus.cloudsim.provisioners.PeProvisioner
  - - org.cloudbus.cloudsim.provisioners.PeProvisionerSimple

- ---

    

  ```
  public class PeProvisionerSimple
  extends PeProvisioner
  ```

  The Class PeProvisionerSimple.

  Since:
  :   CloudSim Toolkit 2.0

- - ### Constructor Summary

    Constructors

    Constructor and Description

    `PeProvisionerSimple(double availableMips)`

    Creates the PeProvisionerSimple object.
  - ### Method Summary

    Methods

    | Modifier and Type | Method and Description |
    | --- | --- |
    | `boolean` | `allocateMipsForVm(java.lang.String vmUid, double mips)` Allocates MIPS for a given VM. |
    | `boolean` | `allocateMipsForVm(Vm vm, double mips)` Allocates MIPS for a given VM. |
    | `boolean` | `allocateMipsForVm(Vm vm, java.util.List<java.lang.Double> mips)` Allocates MIPS for a given VM. |
    | `void` | `deallocateMipsForAllVms()` Releases MIPS used by all VMs. |
    | `void` | `deallocateMipsForVm(Vm vm)` Releases MIPS used by a VM. |
    | `java.util.List<java.lang.Double>` | `getAllocatedMipsForVm(Vm vm)` Gets allocated MIPS for a given VM. |
    | `double` | `getAllocatedMipsForVmByVirtualPeId(Vm vm, int peId)` Gets allocated MIPS for a given VM for a given virtual Pe. |
    | `protected java.util.Map<java.lang.String,java.util.List<java.lang.Double>>` | `getPeTable()` Gets the pe table. |
    | `double` | `getTotalAllocatedMipsForVm(Vm vm)` Gets total allocated MIPS for a given VM for all PEs. |
    | `protected void` | `setPeTable(java.util.Map<java.lang.String,? extends java.util.List<java.lang.Double>> peTable)` Sets the pe table. |

    - ### Methods inherited from class org.cloudbus.cloudsim.provisioners.PeProvisioner

      `getAvailableMips, getMips, getTotalAllocatedMips, getUtilization, setAvailableMips, setMips`
    - ### Methods inherited from class java.lang.Object

      `clone, equals, finalize, getClass, hashCode, notify, notifyAll, toString, wait, wait, wait`

- - ### Constructor Detail


    - #### PeProvisionerSimple

      ```
      public PeProvisionerSimple(double availableMips)
      ```

      Creates the PeProvisionerSimple object.

      Parameters:
      :   `availableMips` - the available mips
  - ### Method Detail


    - #### allocateMipsForVm

      ```
      public boolean allocateMipsForVm(Vm vm,
                              double mips)
      ```

      **Description copied from class: `PeProvisioner`**

      Allocates MIPS for a given VM.

      **Specified by:**
      :   `allocateMipsForVm` in class `PeProvisioner`

      Parameters:
      :   `vm` - virtual machine for which the MIPS are being allocated
      :   `mips` - the mips

      Returns:
      :   $true if the MIPS could be allocated; $false otherwise


    - #### allocateMipsForVm

      ```
      public boolean allocateMipsForVm(java.lang.String vmUid,
                              double mips)
      ```

      **Description copied from class: `PeProvisioner`**

      Allocates MIPS for a given VM.

      **Specified by:**
      :   `allocateMipsForVm` in class `PeProvisioner`

      Parameters:
      :   `vmUid` - the vm uid
      :   `mips` - the mips

      Returns:
      :   $true if the MIPS could be allocated; $false otherwise


    - #### allocateMipsForVm

      ```
      public boolean allocateMipsForVm(Vm vm,
                              java.util.List<java.lang.Double> mips)
      ```

      **Description copied from class: `PeProvisioner`**

      Allocates MIPS for a given VM.

      **Specified by:**
      :   `allocateMipsForVm` in class `PeProvisioner`

      Parameters:
      :   `vm` - virtual machine for which the MIPS are being allocated
      :   `mips` - the mips for each virtual Pe

      Returns:
      :   $true if the MIPS could be allocated; $false otherwise


    - #### deallocateMipsForAllVms

      ```
      public void deallocateMipsForAllVms()
      ```

      **Description copied from class: `PeProvisioner`**

      Releases MIPS used by all VMs.

      **Overrides:**
      :   `deallocateMipsForAllVms` in class `PeProvisioner`


    - #### getAllocatedMipsForVmByVirtualPeId

      ```
      public double getAllocatedMipsForVmByVirtualPeId(Vm vm,
                                              int peId)
      ```

      **Description copied from class: `PeProvisioner`**

      Gets allocated MIPS for a given VM for a given virtual Pe.

      **Specified by:**
      :   `getAllocatedMipsForVmByVirtualPeId` in class `PeProvisioner`

      Parameters:
      :   `vm` - virtual machine for which the MIPS are being allocated
      :   `peId` - the pe id

      Returns:
      :   allocated MIPS


    - #### getAllocatedMipsForVm

      ```
      public java.util.List<java.lang.Double> getAllocatedMipsForVm(Vm vm)
      ```

      **Description copied from class: `PeProvisioner`**

      Gets allocated MIPS for a given VM.

      **Specified by:**
      :   `getAllocatedMipsForVm` in class `PeProvisioner`

      Parameters:
      :   `vm` - virtual machine for which the MIPS are being allocated

      Returns:
      :   array of allocated MIPS


    - #### getTotalAllocatedMipsForVm

      ```
      public double getTotalAllocatedMipsForVm(Vm vm)
      ```

      **Description copied from class: `PeProvisioner`**

      Gets total allocated MIPS for a given VM for all PEs.

      **Specified by:**
      :   `getTotalAllocatedMipsForVm` in class `PeProvisioner`

      Parameters:
      :   `vm` - virtual machine for which the MIPS are being allocated

      Returns:
      :   total allocated MIPS


    - #### deallocateMipsForVm

      ```
      public void deallocateMipsForVm(Vm vm)
      ```

      **Description copied from class: `PeProvisioner`**

      Releases MIPS used by a VM.

      **Specified by:**
      :   `deallocateMipsForVm` in class `PeProvisioner`

      Parameters:
      :   `vm` - the vm


    - #### getPeTable

      ```
      protected java.util.Map<java.lang.String,java.util.List<java.lang.Double>> getPeTable()
      ```

      Gets the pe table.

      Returns:
      :   the peTable


    - #### setPeTable

      ```
      protected void setPeTable(java.util.Map<java.lang.String,? extends java.util.List<java.lang.Double>> peTable)
      ```

      Sets the pe table.

      Parameters:
      :   `peTable` - the peTable to set


- Overview
- Package
- Class
- Use
- Tree
- Deprecated
- Index
- Help

- Prev Class
- Next Class

- Frames
- No Frames

- All Classes

- Summary:
- Nested |
- Field |
- Constr |
- Method

- Detail:
- Field |
- Constr |
- Method
